# A Rare Cause of Ascites-Disseminated TB with Peritonitis in a Middle-Aged Female

**DOI:** 10.1155/2019/5076857

**Published:** 2019-05-26

**Authors:** Sahathevan Vithoosan, Ponnudurai Shanjeeban, Joseph Philip Anpalahan, Paramarajan Piranavan, Harindra Karunatilake, Ananda Jayanaga

**Affiliations:** ^1^National Hospital, Colombo, Sri Lanka; ^2^Internal Medicine, Saint Vincent Hospital, Worcester, MA 01608, USA

## Abstract

**Background:**

Disseminated tuberculosis (TB) has been increasingly recognized in adults in the recent times due to increased prevalence of immune suppression. Here we describe a case of 47-year-old female who presented with disproportionate ascites where the diagnosis of disseminated TB was delayed.

**Case Report:**

A 47-year-old previously healthy female presented with generalised body swelling with disproportionate ascites and loss of appetite and weight for four-month duration. She denied any contact or past history of TB and reported no respiratory symptoms. Physical examination revealed significant ascites. There was no lymphadenopathy or hepatosplenomegaly. Respiratory system examination was normal. Her Erythrocyte Sedimentation Rate (ESR) was above 100. Tuberculin skin test was positive with 17mm. Contrast Enhanced Computed Tomography (CECT) abdomen revealed chronic liver disease with ascites. Diagnostic laparoscopy was in favour of miliary TB and the peritoneal biopsy revealed granulomatous inflammation with caseous necrosis, suggestive of TB. The patient was started on antituberculosis treatment and subsequently improved.

**Conclusion:**

TB peritonitis due to disseminated TB should be considered in the differential diagnosis of disproportionate ascites. Even though the diagnosis is difficult, diagnostic laparoscopy and biopsy is very helpful. It is important to have an early diagnosis since delay in treatment can be detrimental in most cases.

## 1. Introduction

Tuberculosis (TB) is a systemic infectious disease caused by* Mycobacterium tuberculosis* that affects multiple tissues and organs. Because it has diverse presentations, it requires a high index of suspicion. Disseminated TB has been increasingly recognised in adults in recent times. This is due to the increased prevalence of immune suppression due to acquired immunodeficiency syndrome (AIDS) and immunosuppressive therapies for various medical disorders [1]. Tuberculous peritonitis is a rare presentation of disseminated TB [2]. There is usually a delay of about one month from presentation to diagnosis in tuberculous peritonitis [2]. Here, we describe a case of a 47-year-old previously healthy female who presented with disproportionate ascites, where the diagnosis of disseminated TB was delayed. The case highlights the importance of and difficulty in diagnosing disseminated TB.

## 2. Case Presentation

A 47-year-old previously healthy female presented with generalised body swelling, disproportionate ascites, loss of appetite, and loss of weight for four months' duration. She denied any fever, night sweats, yellowish discolouration of the eyes, hematemesis, melena, chronic cough, and haemoptysis. She reported no history of orthopnoea and paroxysmal nocturnal dyspnoea, and her urine output remained normal.

Her past medical history was not significant for any liver, renal, or cardiac disease. She denied any past or contact history of TB. She reported no use of alcohol or herbal medications and no intravenous drug abuse. She was in a monogamous relationship and she denied any family history of liver or renal disease.

She was afebrile on admission and her vitals included a pulse rate within normal limits. On exam, she was emaciated with a body mass index of 18, and she had significant ascites with mild ankle oedema. She was anicteric and did not have any lymphadenopathy, hepatosplenomegaly, or the peripheral stigmata of chronic liver disease. Respiratory system and cardiovascular examinations were within normal limits. On eye exam, there was no evidence of choroid tubercles.

The initial laboratory findings are summarised in Table 1.

The anaemia workup, including serum iron studies, vitamin B12, and folate testing, was normal. A blood picture revealed normochromic normocytic anaemia and thrombocytosis, suggestive of anaemia of chronic disease. Thyroid function tests were normal. She did not have any proteinuria, and her international normalised ratio (INR) was normal. Repeated blood cultures, urine culture, and sputum culture were sterile, and a human immunodeficiency virus (HIV) fourth-generation test was negative. CA 125 was mildly elevated to 175 U/ml (<46 U/ml).

The initial chest x-ray (CXR) was normal, and her transthoracic two-dimensional echocardiography showed normal systolic and diastolic functions and no evidence of constrictive pericarditis. A transvaginal ultrasound (US) did not reveal any ovarian pathology. Meanwhile, an abdomen US revealed a mildly hyperechoic liver with moderate ascites. Contrast Enhanced Computed Tomography (CECT) of the abdomen and pelvis revealed a normal-sized liver with evidence of heterogenicity of the liver parenchyma with minimal nodularity of the liver surface and moderate ascites with no portal vein thrombosis or lymphadenopathy. The spleen, gallbladder, biliary tract, and intestines were normal, as were the ovaries. The findings were in favour of possible decompensated chronic liver disease (CLD). However, the CLD workup did not reveal any potential aetiology (Table 2). Upper gastrointestinal endoscopy (UGIE) was normal and did not show any evidence of portal gastropathy or varices.

The initial diagnosis of possible cryptogenic CLD was made, and the patient was started on spironolactone and furosemide. Even though the diagnostic paracentesis was planned on admission, it was delayed due to the patient's refusal, but we were to do initiate after a few days. The analysis of the ascitic fluid revealed an exudative picture. There was no evidence of spontaneous bacterial peritonitis, and the ascitic fluid cytology was negative for malignant cells.

With elevated inflammatory markers and an exudative type of ascites, we decided to proceed with further investigations to rule out possible peritoneal TB. The ascitic fluid acid-fast bacilli (AFB) test was negative. The ascitic fluid bacterial, TB culture, and TB Gene Xpert were negative. The tuberculin sensitivity test (Mantoux) was positive at 17 mm without ulceration. Her repeated sputum for AFB, TB culture, and GeneXpert were negative.

**Figure 1:**
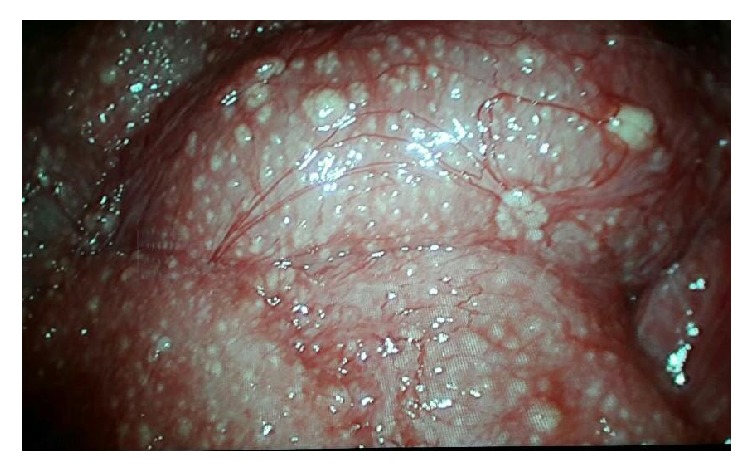
Studded miliary tubercles in laparoscopic examination.

Because the clinical picture did not fit the imaging findings, we decided to proceed with diagnostic laparoscopy and peritoneal biopsy. The laparoscopy was in favour of miliary TB (Figure 1) and the peritoneal biopsy revealed granulomatous inflammation with caseous necrosis favouring TB. She was started on anti-TB treatment (ATT), and on initial follow-up, she showed improvement in her clinical, biochemical, and radiological parameters. Her fever settled with ATT, and her ESR was 15 mm/1st hour and CRP was 12 mg/l at 3 weeks after the initiation of ATT.

## 3. Discussion

Tuberculous peritonitis is a rare presentation of extra-pulmonary TB. The diagnosis is difficult due to its presentations overlapping with other diseases [2]. The delayed diagnosis leads to a delay in initiating treatment, which worsens outcomes [3]. A high index of suspicion is important for early diagnosis. Abdominal TB can involve the gastrointestinal tract, peritoneum, lymph nodes, or solid organs [4], and it comprises 5% of all cases of TB. Peritoneal TB mostly occurs following the reactivation of latent TB foci in the peritoneum established via the bloodstream and spread from a primary lung focus. Both the visceral and parietal peritonea can be studded with tubercles. Clinical manifestations of peritoneal TB include ascites, abdominal pain, and fever [3]. Three different forms of peritoneal TB have been reported, which include wet TB with ascites, dry type with adhesions, and fibrotic type with omental thickening and loculated ascites [5].

Our patient's ascites were disproportionate to the peripheral oedema. She was investigated for the potential causes for disproportionately predominant ascites, which include cirrhosis with portal hypertension, ovarian carcinoma, constrictive pericarditis, and peritonitis. Other causes of generalised oedema, such as congestive heart failure, nephrotic syndrome, and renal impairment, were also excluded by history, physical examination, and basic investigations. Both the abdominal US and the abdominal CECT revealed CLD with ascites. However, she did not have the peripheral stigmata of CLD and did not have significant risk factors for CLD. Blood investigations revealed an elevated platelet count and a normal INR. The absence of oesophageal varices and portal gastropathy further challenged the diagnosis of CLD. The exudative type of fluid on an ascitic fluid analysis led to the suspicion of any form of peritonitis in our patient. She did not have the typical constitutional symptoms or the risk factors for TB. Her CXR was normal, but her tuberculin sensitivity test (TST) was positive. However, the TST is of limited value in clinical work, especially in countries with a high prevalence of TB and prior Bacillus Calmette-Guerin vaccination. A positive test only indicates the infection but not the presence or extent of tuberculous disease. In our patient, the laparoscopic peritoneal biopsy revealed a peritoneum studded with tubercles, confirming our diagnosis.

It is important to note that the absence of CXR findings does not rule out the possibility of extra-pulmonary TB, as less than 25% of cases with extra-pulmonary TB were found to have an abnormal CXR [5]. The abdominal US can be a useful tool in the investigation for peritoneal TB, which may reveal ascites, lymphadenopathy, bowel wall thickening, and the pseudo kidney sign [5]. Interestingly, our patient's imaging was reported as decompensated CLD.

The ascitic fluid ADA and interferon activity were not checked in our patient due to limited resources. The ascitic fluid ADA is a potentially useful test, which can help when there is a diagnostic dilemma [6]. It was found to be a sensitive test, even in patients with concomitant cirrhosis. However, it must be noted that malignant ascites can give rise to a falsely high ADA [6]. High interferon activity is also noted in TB ascites. Combining ascitic fluid interferon activity with ADA may further increase the sensitivity of TB diagnosis [5]. The ascitic fluid AFB is often negative in cases of peritoneal TB [7]. Even though TB PCR is a useful diagnostic tool, it also has low sensitivity [8].

Our patient's serum CA 125 level was moderately elevated, but further investigations ruled out ovarian pathologies. Although CA 125 is used as a biomarker in the early detection of ovarian carcinoma, there have also been reports of elevated levels of soluble CA 125 in a number of other malignant conditions, such as breast cancer, mesothelioma, non-Hodgkin's lymphoma (NHL), gastric cancer, and leiomyosarcoma of gastrointestinal origin. CA 125 levels have also been found elevated in benign conditions, such as endometriosis, pregnancy, ovulatory cycles, liver diseases, and congestive heart failure, as well as in infectious disease, such as TB peritonitis [9]. There were instances where TB peritonitis has been mistaken as ovarian carcinomatosis based on elevated CA 125 [10]. Localizing CA 125 around tuberculous granuloma using immunohistochemistry was previously reported in literature [11]. Furthermore normalization of CA 125 levels after ATT supports the fact that the raised level could be due to peritoneal TB. So serial measurement of serum CA125 level in TB peritonitis may be of significance in follow-up and monitoring the response of disease to ATT [11].

Reduction in CRP levels after ATT is well described before in several cohorts of tuberculous patients. In one study when CRP levels decrease ≤55% at 2 weeks, it predicted death or hospitalization, with a negative predictive value of 99% [12]. Failure to see a decline after ATT may suggest alternative diagnosis or drug-resistant tuberculosis [13].

In our patient ALP, GGT and bilirubin were significantly elevated compared to other transaminases. The correlation between liver function tests and the site of liver disease in any infiltrative or infective diseases, including TB, was previously reported in the literature. The involvement of the liver parenchyma is usually reflected in elevated transaminases, while the involvement of the biliary tract or porta is usually reflected in elevated ALP and GGT. Bilirubin may be elevated in hepatic TB or miliary TB [14].

The visual appearance during laparoscopy will help in the diagnosis of tuberculous peritonitis [5], and our patient was studded with multiple yellow white tubercles.

Biopsies of the peritoneal lesion, lymph nodes, or other intra-abdominal locations are often necessary for the diagnosis. The laparoscopic biopsy is preferred, as it is minimally invasive and allows for the visualisation of the peritoneum [7]. The biopsy has a diagnostic yield of more than 80% in the diagnosis of tuberculous peritonitis [3]. Our patient's biopsy of the peritoneal lesion revealed granulomatous inflammation with caseous necrosis.

## 4. Conclusion

Tuberculous peritonitis due to disseminated TB should be considered in the differential diagnosis of disproportionate ascites. Even though the diagnosis is difficult, diagnostic laparoscopy and biopsy are highly helpful. It is important to reach an early diagnosis, as delays in treatment can be detrimental in these cases.

## Figures and Tables

**Table 1:** The initial biochemical investigations.

Investigation	Result	Normal range
White cell count(10^9^/L)	10.6	4-11
Neutrophil percentage	67%	
Lymphocyte percentage	28%	
Haemoglobin	7.4	11-13
Platelet count(10^9^ /L)	511	150-450
C- Reactive Protein (CRP)(mg/L)	128	0-6
ESR( mm/1^st^ hour)	110	<10
Serum creatinine(*µ*mol/L)	52	60-120
Serum sodium (mmol/L)	135	135-148
Serum potassium(mmol/L)	4.2	3.5-5.1
Aspartate Transaminase(AST) (U/L)	56	<40
Alanine Transaminase (ALT) (U/L)	44	<40
Alkaline Phosphatase (ALP) (U/L)	776	30-120
Gamma Glutamyl Transferase (GGT)(U/L)	500	<50
Total bilirubin (mg/dl)	6.6	<1.2
Direct bilirubin (mg/dl)	3.4	<0.3
Serum total proteins(g/L)	58	61-80
Serum Albumin(g/L)	25	36-50
Serum Globulin(g/L)	33	22-40

**Table 2:** Aetiological work-up for CLD.

Investigation	Result
*Viral markers*	
Hepatitis B surface antigen Hepatitis C antibody	Negative Negative

*Autoimmune markers*	
ANA Anti smooth muscle antibody	Negative Negative

*Iron studies*	
Total binding capacity Serum iron Serum ferritin	252 mcg/dl(240-450) 60*µ*g/dl (50-170) 310 ng/ml( 12-150)

Serum Ceruloplasmin	28 mg/dl(20-35)
